# Association between amygdala neurokinin-1 receptor availability and anxiety-related personality traits

**DOI:** 10.1038/s41398-018-0163-1

**Published:** 2018-08-28

**Authors:** Johanna M. Hoppe, Andreas Frick, Fredrik Åhs, Clas Linnman, Lieuwe Appel, My Jonasson, Mark Lubberink, Bengt Långström, Örjan Frans, Lars von Knorring, Mats Fredrikson, Tomas Furmark

**Affiliations:** 1 Department of Psychology, Uppsala University, Uppsala, Sweden; 2 Department of Psychology, Stockholm University, Stockholm, Sweden; 3 Department of Clinical Neuroscience, Karolinska Institutet, Stockholm, Sweden; 4 Department of Anesthesiology, Perioperative and Pain Medicine, Boston Children’s Hospital, and Department of Psychiatry, Massachusetts General Hospital, Harvard Medical School, Boston, MA USA; 5 Nuclear Medicine and PET, Department of Surgical Sciences, Uppsala University, Uppsala, Sweden; 6 Medical Physics, Uppsala University Hospital, Uppsala, Sweden; 7 Department of Chemistry, Uppsala University, Uppsala, Sweden; 8 Department of Neuroscience, Psychiatry, Uppsala University, Uppsala, Sweden

## Abstract

Animal studies indicate that substance P (SP) and its preferred neurokinin-1 (NK1) receptor modulate stress and anxiety-related behavior. Alterations in the SP-NK1 system have also been observed in human anxiety disorders, yet little is known about the relation between this system and individual differences in personality traits associated with anxiety propensity and approach-avoidance behavior, including trait anxiety, neuroticism, and extraversion. Exploring this relation could provide important insights into the neurobiological underpinnings of human anxiety and the etiology of anxiety disorders, as anxious traits are associated with increased susceptibility to develop psychopathological conditions. Here we examined the relationship between central NK1 receptor availability and self-rated measures of trait anxiety, neuroticism, and extraversion. The amygdala was chosen as the primary region of interest since this structure has been suggested to mediate the effect of the SP-NK1 system on anxiety. Anxious traits and NK1 receptor availability, determined with positron emission tomography and the radiotracer [^11^C]GR205171, were measured in 17 healthy individuals. Voxel-wise analyses showed a significant positive correlation between bilateral amygdala NK1 receptor availability and trait anxiety, and a trend in similar direction was observed for neuroticism. Conversely, extraversion was found to be negatively associated with amygdala NK1 receptor availability. Extraversion also correlated negatively with the NK1 measure in the cuneus/precuneus and fusiform gyrus according to exploratory whole-brain analyses. In conclusion, our findings indicate that amygdala NK1 receptor availability is associated with anxiety-related personality traits in healthy subjects, consistent with a modulatory role for the SP-NK1 system in human anxiety.

## Introduction

The neuropeptide substance P (SP)^1^ and its preferred neurokinin-1 receptor (NK1)^2^ are abundantly distributed in the mammalian nervous system, including structures in the brain's fear network such as the amygdala^3,4^. Animal studies provide evidence that the SP-NK1 system modulates stress and anxiety-related behavior^3,5^ and alterations in this system have also been suggested to be involved in human anxiety disorders^6–8^. For instance, patients with social anxiety disorder (SAD), as compared to healthy subjects, have increased NK1 receptor availability in the right amygdala^9^ and treatment with the NK1 receptor antagonist GR205171 has been shown to alleviate social anxiety in parallel with decreased regional cerebral blood flow in the amygdala during public speaking^10^. Also, in a positron emission tomography (PET) study of patients with specific phobia, exposure to phobic, as compared to non-phobic, stimuli was associated with a reduced uptake of the radiotracer [^11^C]GR205171 in the amygdala, putatively reflecting increased release of endogenous SP^11^. Moreover, elevated cerebrospinal fluid SP concentration and increased amygdala NK1 receptor availability have been observed in patients with posttraumatic stress disorder, compared with healthy subjects^12,13^. Notably, whereas beneficial effects of NK1 receptor antagonists were observed initially in experimental and clinical studies, these effects could not be confirmed in phase III clinical trials^10,14–16^. However, despite these mixed results, converging findings from several studies indicate that the SP-NK1 system is part of the neural underpinnings of anxiety disorders^9–13^. Furthermore, it has been argued that the negative results from phase III trials could be influenced by several factors, such as use of inadequate drug dose range, use of NK1 receptor antagonist drugs with insufficient pharmacokinetic and pharmacodynamic properties, as well as heterogeneous patient selection^8,17^. In sum, as the role of the SP-NK1 system in human anxiety is still not fully understood, further knowledge of this system is important to understand the neurobiological basis of psychopathological conditions.

Only a few studies have explored the role of the SP-NK1 system in anxiety in healthy subjects^18–21^. Thus, it is not known if alterations in the SP-NK1 system occur primarily in psychiatric disorders, or if they extend also to personality traits associated with anxiety propensity and approach-avoidance behavior, such as trait anxiety^22^, neuroticism and the extraversion–introversion dimension^23^. Even though there are studies demonstrating that administration of NK1 receptor antagonists affects some aspects of anxiety-related emotional processes in healthy subjects^18–20^, the relation between individual differences in anxious personality traits and the neurochemical properties of the SP-NK1 system is incompletely understood.

Anxiety-related personality traits are associated with poorer quality of life, increased susceptibility to develop psychopathological states^24–26^ and they impose high societal cost^27^. Further knowledge of their etiology is therefore essential. A biological basis for personality traits has been suggested^28^, and individual differences in trait anxiety, neuroticism and extraversion have been related to alterations in brain function, including altered amygdala reactivity and connectivity^29–31^. Animal models of anxiety^32,33^ indicate that the SP-NK1 system may be involved in the neural underpinnings of anxiety-related traits. For example, rats bred to exhibit highly anxious behavior, show increased stress-induced release of SP in the medial amygdala in comparison to their low anxiety counterpart^34^. Also, the response to NK1 receptor antagonists is modulated by differences in affective temperament, altering anxious behavior only in rats with high trait anxiety^35^. Furthermore, pointing towards a relation between the SP-NK1 system and extraverted behavior, acute administration of NK1 receptor antagonists has been observed to increase the duration of social contact in gerbils^36,37^. Collectively, these results suggest that the SP-NK1 system is involved in the propensity for anxiety and approach-avoidance behavior. Individual differences in the SP-NK1 system could therefore play a role not only in psychiatric disorders, but also more generally in anxiety-related personality traits.

The aim of the present study was to evaluate the association between the SP-NK1 system and anxious traits in healthy volunteers. We used PET and the highly selective NK1 receptor antagonist GR205171^38^ labeled with [^11^C] as a radioligand^39,40^ to examine the relationship between NK1 receptor availability in the amygdala and self-report measures of trait anxiety, neuroticism, and extraversion^23^. The amygdala was selected as the primary region of interest since previous studies suggest that this structure modulates the effect of the SP-NK1 system on anxiety both in animals and in humans^9–11,20,41–43^ and because amygdala reactivity and connectivity are related to anxious traits^30,31,44–46^. Exploratory whole-brain analyses were, however, also performed. We hypothesized that trait anxiety and neuroticism would be positively associated with NK1 receptor availability in the amygdala, reflecting a presumed anxiogenic effect of the SP-NK1 system. Conversely, we predicted a negative association between amygdala NK1 receptor availability and the degree of extraversion, i.e., higher availability in less extraverted individuals.

## Materials and methods

### Participants

In total 18 healthy subjects were recruited through advertising. Participants underwent a medical examination, and the Mini International Neuropsychiatric Interview^47^ was performed by a psychiatrist to exclude current or previous history of psychiatric disorder according to DSM-IV. Exclusion criteria were alcohol/drug abuse, neurological disorder, somatic disease, chronic use of prescription medication, left handedness, previous PET examination, family history of cancer, pregnancy and menopause. Due to technical problems, PET data from one participant could not be analyzed, leaving a sample of 17 participants (M ± SD age: 34.7 ± 9.8 years; 9 women). There were no significant differences in age between men and women (*t*(15) = 0.59, *P* *=* 0.56). The current sample has been described previously, e.g., in comparison to patients with social anxiety disorder^9^.

The study was approved by the Uppsala University Medical Faculty Ethical Review Board and the Radiation Ethics Committee at Uppsala University Hospital. Written informed consent was obtained from all participants before the study start.

### Assessment of anxiety-related traits

Trait anxiety was assessed with the Spielberger State-Trait Anxiety Inventory (STAI-T)^22^ and the personality traits neuroticism and extraversion, were measured using the self-rated version of the Revised NEO Personality Inventory (NEO PI-R)^23^.

### Radioligand

[^11^C]GR205171 is a highly selective NK1 receptor antagonist radiotracer with high affinity and good blood-brain barrier penetration^38–40^. This radiotracer shows slow dissociation from the NK1 receptor and has therefore been suggested to be suitable for assessments of NK1 receptor density^39^.

### PET image acquisition and preprocessing

Image acquisition was performed using an ECAT Exact HR + PET scanner (Siemens/CTI, Knoxville, TN, USA) with an axial field of view of 155 mm. Subjects were instructed to fast for 3 h and refrain from alcohol, caffeine, and tobacco 12 h before the PET-investigation. Subjects were positioned supine in the PET scanner with their heads gently fixated and a venous catheter was inserted. Each PET-investigation commenced with a 10-min transmission scan using 3 retractable germanium (^68^Ge) rotating line sources.

The radiotracer [^11^C]GR205171 was injected intravenously as a fast bolus simultaneously with the start of the emission scan. Data were acquired in three-dimensional (3D) mode and consisted of 17 frames (4 × 60 s, 3 × 120 s, 10 × 300 s) with a total duration of 60 min. Participants were scanned in the resting state and received on average 398 (SD: 11) MBq, equal to 6 (SD: 1) MBq/kg body weight. To facilitate spatial normalization, a [^15^O]water PET scan was acquired (3 frames × 30 s) with administration of ~10 MBq/kg body weight during the resting state. Dynamic images were reconstructed using ordered subset expectation maximization with six iterations and eight subsets and a 4 mm Hanning post-filter^9^.

Parametric [^11^C]GR205171 images showing influx rate K_i_ (ml cm^−3^ min^−1^) for each voxel, indexing NK1 receptor availability, were generated applying reference Patlak graphical analysis^48,49^ using a time interval of 30–60 min depicting irreversible tracer binding. The cerebellum was chosen as the reference region as it displays a paucity of NK1 receptors^39,50,51^. Cerebellum was defined using the co-registered [^15^O]water PET scan of each participant and the PVElab software^52^, an observer-independent probabilistic approach for automatic generation of volumes of interest, and transferred to the dynamic [^11^C]GR205171 image to obtain a cerebellum time–activity curve.

Each individual’s [^11^C]GR205171 K_i_ image was co-registered to their [^15^O]water summation image using affine transformation. The [^15^O]water summation image was then normalized to the PET template from Statistical Parametric Mapping 8 (SPM8; Wellcome Department of Cognitive Neurology, University College London, www.fil.ion.ucl.ac.uk), and the calculated transformation parameters were applied to the [^11^C]GR205171 K_i_ image, resulting in [^11^C]GR205171 K_i_ images normalized to the Montreal Neurological Institute (MNI) standard space with isotropic 2 × 2 × 2 mm^3^ voxels. The MNI normalized images were subsequently smoothed with a 12 mm isotropic Gaussian kernel to enhance the signal to noise ratio.

### Statistical analysis

Participant characteristics and behavioral data were analyzed using IBM SPSS Statistics for Windows, version 22 (IBM Corp., Armonk, NY), whereas statistical analyses of PET data were conducted using SPM8 (Welcome Department of Cognitive Neurology, University College London, www.fil.ion.ucl.ac.uk). The amygdala was chosen as the primary region of interest (ROI) based on previous findings suggesting that this structure mediates the effect of the SP-NK1 system on anxiety^9–11,20,41–43^. The Automated Anatomical Labeling library from the Wake Forest University Pickatlas^53^ was used to define the amygdala, i.e., the right and left amygdala ROIs were included jointly in all analyses. Exploratory whole-brain analyses were performed in addition to ROI analyses. Age and sex were entered as covariates in all analyses since NK1 receptor availability has been suggested to be moderated by these variables^54,55^. For ROI analyses, the statistical threshold for significance was set at *P* *<* 0.05 family wise error (FWE) corrected for multiple comparisons, and exploratory whole-brain analyses were additionally performed using a combined criterion with the uncorrected statistical threshold set at *P* < 0.001 at the voxel level and *P* *<* 0.05 for cluster extent.

To explore the relationship between trait anxiety, neuroticism, extraversion, and NK1 receptor availability within the amygdala, parametric [^11^C]GR205171 K_i_ images were entered into three separate regression models using the total scores for STAI-T and NEO PI-R subscales for neuroticism and extraversion as predictors. The same analyses were also performed using the extracted mean [^11^C]GR205171 K_i_ value within the bilateral amygdala, i.e., the same ROI used in previous analyses. Additionally, in order to assess the specific associations between NK1 receptor availability and each of the studied anxiety-related traits, parametric [^11^C]GR205171 K_i_ images were entered into a multiple regression with the total scores for all traits, i.e., STAI-T, neuroticism and extraversion, as predictors. Moreover, to further evaluate the association between amygdala NK1 receptor availability and anxious traits, the total scores for each trait were also entered into a principal component analysis (PCA), extracting scores for each participant on two principal components, and subsequently assessing their correlation with mean amygdala NK1 receptor availability. Furthermore, regression models with the remaining NEO PI-R factors, i.e., openness, agreeableness and conscientiousness, were conducted to control for associations between NK1 availability and non-anxious personality traits. Finally, ratings of state anxiety experienced during the PET scan, as measured with STAI-S^22^, were entered as a covariate of no interest in the STAI-T regression model to control for the influence of state anxiety on the association between NK1 receptor availability and trait anxiety.

## Results

Descriptive statistics and correlations for personality traits are listed in Table 1. Whereas trait anxiety and neuroticism were positively correlated, extraversion was not significantly associated with any of the other studied traits. The present sample had mean scores that were numerically somewhat lower for neuroticism and higher for extraversion, as well as showed lower variance in extraversion scores, in comparison to the Swedish normative group for NEO PI-R (neuroticism: M = 78.0, SD = 22.5; extraversion: M = 107.6, SD = 20.7)^56,57^.

**Table 1:** Descriptive statistics and correlations of self-report data for trait anxiety (STAI-T), neuroticism and extraversion as measured with NEO-PI-R

	Trait anxiety	Neuroticism	Extraversion
Descriptive statistics			
Mean	29.1	62.8	126.5
SD	7.0	23.9	13.4
Range	20–45	36–122	101–154
Correlations^a^			
Trait anxiety	1	0.72	−0.06
		*P* = 0.001	*P* = 0.82
Neuroticism	—	1	−0.15
			*P* = 0.57
Extraversion	—	—	1

^a^Pearson correlation coefficient

**Fig. 1:**
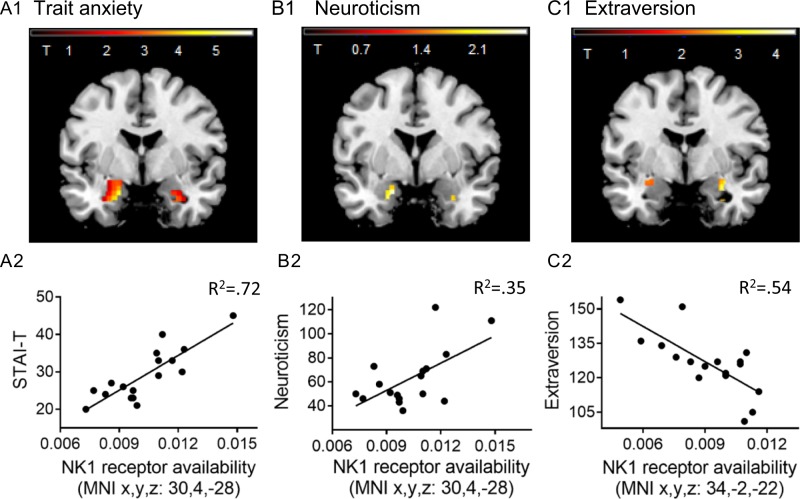
Results from ROI-based statistical parametrical mapping overlaid on a standard brain (A1, B1, and C1) and corresponding regression plots (A2, B2, C2) showing the association between anxiety-related traits and amygdala NK1 receptor availability. Images are displayed at *P* < 0.05 uncorrected overlaid on a template MR image, for illustrative purposes. Regression plots and R-Square values (*p* < 0.05) are based on the peak voxel associated with each trait

Age and sex corrected statistical parametric mapping revealed a positive association between NK1 receptor availability in the bilateral amygdala and trait anxiety as measured with STAI-T (Table 2, Fig. 1). We also found a trend towards a positive association between neuroticism and NK1 receptor availability bilaterally in the amygdala (right: MNI *x*, *y*, *z*: 30, 4, −28; *Z* = 2.34, *P*_uncorr_ = 0.010, 152 mm^3^; left: MNI *x*, *y*, *z*: −18, −2, −22; *Z* = 2.25, *P*_uncorr_ = 0.012, 208 mm^3^). Extraversion was negatively associated with NK1 receptor availability in the right amygdala (Table 2, Fig. 1), and at an uncorrected p-level in the left amygdala (MNI *x*, *y*, *z*: -28, −8, −12; *Z* = 2.48, *P*_uncorr_ = 0.007, 240 mm^3^). Mean NK1 receptor availability ([^11^C]GR205171 K_i_) within the bilateral amygdala was also positively associated with STAI-T (*r* = 0.59, *P* = 0.010) and negatively with extraversion (*r* = −0.44, *P* = 0.049).

**Table 2:** Statistical parametric mapping of significant associations between amygdala NK1 receptor availability, trait anxiety, and extraversion in healthy subjects

NK1 receptor availability	*Z*	*P*_*FWE*_	Volume^a^	*x*	*y*	*z* ^b^
Positive association						
Trait anxiety						
Right amygdala	4.10	0.002	112	30	4	−28
Left amygdala	3.56	0.010	176	−18	−2	−22
Negative association						
Extraversion						
Right amygdala	3.25	0.026	64	34	−2	−22

^a^Volume in mm^3^, voxel size: 8 mm^3^

^b^Peak voxel coordinates in MNI (Montreal Neurological Institute) space

The associations between NK1 receptor availability, trait anxiety (right: MNI *x*, *y*, *z*: 32, 4, −26; *Z* = 3.64, *P*_*FWE*_ = 0.010, 144 mm;^3^ left: MNI *x*, *y*, *z*: −26, 0, −28; *Z* = 3.39, *P*_*FWE*_ = 0.021, 72 mm^3^) and extraversion (right: MNI *x*, *y*, *z*: 32, −2, −22; *Z* = 3.67, *P*_*FWE*_ = 0.009, 288 mm^3^), remained significant also when including all traits as predictors in a single multiple regression analysis. No significant association was observed between NK1 receptor availability and neuroticism in the multiple regression. However, when removing STAI-T scores from the model, controlling only for extraversion, a trend towards a positive association between neuroticism and NK1 receptor availability was observed bilaterally in the amygdala (right: MNI *x*, *y*, *z*: 30, 4, −28; *Z* = 2.19, *P*_uncorr_ = 0.014, 96 mm;^3^ left: MNI *x*, *y*, *z*: −18, 0, −22; *Z* = 2.09, *P*_uncorr_ = 0.018, 120 mm^3^). The association between amygdala NK1 receptor availability and trait anxiety remained significant also when controlling for rating of state anxiety during the PET scan (STAI-S: M = 25.6, SD = 5.2; right: MNI *x*, *y*, *z*: 30, 4, −28; *Z* = 3.77, *P*_*FWE*_ = 0.006, 88 mm;^3^ left: MNI *x*, *y*, *z*: −18, −2, −22; Z = 3.83, *P*_*FWE*_ = 0.046, 8 mm^3^). No association was found between state anxiety (STAI-S) scores and NK1 receptor availability.

Results from the PCA performed on the total scores from the anxious traits revealed one principal component (PC1) comprising trait anxiety and neuroticism with loadings of 0.91 and 0.92 respectively, whereas extraversion loaded strongly (0.97) on a second component (PC2). PC1 and PC2 explained 58% and 33% respectively of the variance in anxiety-related traits. Subsequent bivariate correlation analyses showed a positive association between mean amygdala NK1 receptor availability and scores on PC1, i.e., trait anxiety and neuroticism (*r* = 0.49, *p* = 0.048), but no significant association was observed with scores on PC2, i.e., extraversion (*r* = −0.30, *p* = 0.25).

Exploratory whole-brain analysis showed no significant associations, within any brain region, between NK1 receptor availability and trait anxiety or neuroticism, respectively. However, a negative association was found between extraversion and NK1 receptor availability within the right precuneus/cuneus (MNI *x*, *y*, *z*: 10, −84, 38; *Z* = 4.68, *P*_uncorr_ < 0.001, 2240 mm^3^, Brodmann area 19 and MNI *x*, *y*, *z*: 10, −72, 20; *Z* = 3.93, *P*_uncorr_ < 0.001, 3600 mm^3^, Brodmann area 31) and left fusiform gyrus (MNI *x*, *y*, *z*: −28, −68, −10; *Z* = 4.30, *P*_uncorr_ < 0.001, 1856 mm^3^, Brodmann area 19).

NK1 receptor availability was not significantly associated with any of the remaining NEO PI-R scales, i.e., openness, agreeableness, or conscientiousness, in the amygdala or any other brain region.

## Discussion

Our aim was to evaluate the association between NK1 receptors and anxiety-related traits in healthy subjects. The results were in line with our hypotheses, showing a positive association between amygdala NK1 receptor availability and trait anxiety as well as a negative association between the amygdala NK1 measure and extraversion. NK1 receptor availability and neuroticism were also positively correlated, but only at statistical trend level. In addition, our results suggest that NK1 receptor availability may be specific to anxiety-related traits, since no association was observed with either of the remaining non-anxious NEO PI-R personality traits. Thus, we demonstrate that various anxiety-related traits are linked to amygdala NK1 receptor availability in a non-psychiatric population, consistent with previous findings indicating a role for the SP-NK1 system in human anxiety^9–13,18–20^.

The positive association between trait anxiety and amygdala NK1 receptor availability supports previous observations of an anxiogenic role of the SP-NK1 system^9–13^, and it is in good agreement with studies showing that the effect of the SP-NK1 system on anxiety is mediated by the amygdala^41–43^. Even though the relation between amygdala NK1 availability and neuroticism only emerged at a statistical trend level, the association was in the hypothesized direction, i.e., same as for trait anxiety (see Fig. 1). Furthermore, our findings are consistent with imaging studies showing that trait anxiety and neuroticism are associated with altered amygdala function^31,44,45,58^. Thus, it could be speculated that the relation between these traits and amygdala function involves the SP-NK1 system.

Extraversion is associated with the propensity for approach behavior and experiencing positive affect^23,59^. As predicted, we noted a negative correlation between this personality trait and amygdala NK1 receptor availability, i.e., higher availability in the less extraverted individuals. Our results are consistent with findings showing enhanced amygdala NK1 receptor availability in patients with social anxiety disorder^9^, a psychopathological condition negatively associated with extraversion^24^. The negative association between amygdala NK1 receptor availability and extraversion is also in line with observations from animal studies showing that blockage of this receptor, by administration of NK1 receptor antagonists, increases the duration of social contact in gerbils^36,37^. Taken together, our results and previous findings^9,10,36,37^, suggest that the SP-NK1 system could potentially mediate extraverted behavior.

Exploratory whole-brain analyses further showed a negative association between extraversion and NK1 receptor availability in visual areas (cuneus and fusiform gyrus), i.e., the same direction as for the amygdala. Extraversion has previously been related to differential neural processing of positive visual stimuli, such as enhanced amygdala reactivity in response to happy facial expressions^46,60^ and alterations in visual attention to emotional stimuli in parallel with activation of the fusiform gyrus^61^. Supporting a relation between the SP-NK1 system and extraversion, NK1 receptor antagonists have been shown to influence processing of visual stimuli of positive emotional valence^18–20^. For instance, a single dose of the NK1 receptor antagonist aprepitant (125 mg) improves recognition of, and alters the amygdala response to happy facial expressions^19,20^. In addition to visual areas, extraversion was found to be negatively associated with NK1 receptor availability within the precuneus, a structure implicated in complex tasks such as self-centered mental imagery and experience of agency^62^. Consistent with our findings, McCabe and colleagues demonstrated that acute administration of NK1 receptor antagonists in healthy subjects increased the activity within the precuneus during presentation of positive vs. neutral words in an emotional counting Stroop test^20^. However, our exploratory whole-brain analyses did not reveal any additional significant associations for either trait anxiety or neuroticism. Perhaps the association between the SP-NK1 system and these negative affectivity traits are predominantly modulated by the amygdala. Alternatively, the relation in other regions could not be detected due to the restricted sample size, limiting statistical power.

Studying the role of the SP-NK1 system in anxiety-related traits in healthy subjects may provide important insights into the neurobiological underpinnings of human anxiety and the etiology of anxiety disorders. For instance, our findings suggest that the increased susceptibility to develop psychopathological conditions related to anxious traits^24–26^ could, at least in part, be mediated by individual variation in the SP-NK1 system. This system could influence anxious traits by modulating negative affectivity directly, but also by altering the propensity for approach–avoidance behavior^3,36,37,42^. Consistent with our findings, previous results^18–20^ indicate that the SP-NK1 system may also influence anxious traits by affecting processing of positively valenced stimuli. In sum, our findings emphasize the need for further knowledge about the SP-NK1 system in human anxiety.

Several limitations of the present study need to be considered. First, our results are based on a limited number of participants and should be regarded as preliminary. A priori power analysis was not performed, since the study was conducted on existing PET-data from subjects serving as healthy controls in a clinical trial^9^, but post-hoc power analyses, based on correlation coefficients between mean NK1 receptor availability in the bilateral amygdala and each trait (α = 0.05), indicated that the power to detect significant effects was 78%, 15%, and 46% for trait anxiety, neuroticism, and extraversion, respectively. Also, even though our findings suggest that the SP-NK1 system modulates anxiety-related traits, a prior study on patiens with personality disorder found no correlations between cerebrospinal fluid SP-like immunoreactivity and neuroticism or extraversion, but a significant relationship with aggression^63^. Thus, our findings need to be replicated with an adequately powered design. Moreover, the correlational approach and lack of genotyping precluded us from drawing any conclusions about the cause of the association between NK1 receptor availability and anxious traits, e.g., differences in genotype^64^ or exposure to life stress^65^.

The high correlation observed between trait anxiety and neuroticism and the results from the PCA, implies that these traits are indeed overlapping constructs^22,23^, and it is not clear why NK1 receptor availability was more strongly associated with trait anxiety than neuroticism. However, although related, these constructs are not identical. Neuroticism consists of six facets, (anxiety, angry hostility, depression, self-consciousness, impulsiveness and vulnerability to stress)^23^ and thus entails a broader construct than trait anxiety, as measured with STAI-T^22^. Moreover, results from the multiple regression analysis indicated that there is a specific association between NK1 receptor availability and trait anxiety, as the results remained significant also when controlling for neuroticism. The unexpected finding that a relation between NK1 receptor availability and neuroticism was only observed at a trend level could potentially be attributed to the limited sample size.

In the current study [^11^C]GR205171 K_i_ is considered to reflect NK1 receptor availability, as this radiotracer has been suggested to be suitable for assessment of receptor density because of its high affinity and specificity for the NK1 receptor, as well as slow tracer-dissociation^39^. Although, NK1 receptor availability may be influenced by endogenous SP levels, either by direct competition with the radioligand^66^ or via receptor internalization^67^. For instance, a study on specific phobia, showed that exposure to phobic, as compared to non-phobic, stimuli was associated with reduced uptake of [^11^C]GR205171 in the amygdala, putatively reflecting increased fear-related release of endogenous SP^11^. The present study, however, was performed during a passive non-stress condition and should be less influenced by fear-related changes in endogenous SP levels. Indeed, measures of state anxiety during the PET image acquisition were low and we found no relation between state anxiety levels and NK1 receptor availability during PET imaging. Accordingly, follow-up analyses showed that the association found between NK1 receptor availability and trait anxiety remained significant after controlling for state anxiety-ratings during the PET scan, and therefore it is not likely that the results could be attributed to differences in state-anxiety. However, we cannot exclude the possibility that our findings may reflect lower endogenous amygdala SP levels in anxious individuals in non-stressed conditions.

In conclusion, we demonstrate that amygdala NK1 receptor availability correlates positively with trait anxiety and negatively with extraversion. Thus, the current study indicates that the SP-NK1 system has an essential role both in modulating anxiety in psychiatric disorders and anxiety-related personality traits in healthy individuals.
